# The curious case of vacuolar ATPase: regulation of signaling pathways

**DOI:** 10.1186/s12943-018-0811-3

**Published:** 2018-02-15

**Authors:** Sahithi Pamarthy, Arpita Kulshrestha, Gajendra K. Katara, Kenneth D. Beaman

**Affiliations:** 1 Department of Microbiology and Immunology, Rosalind Franklin University of Medicine and Science, 3333 Green Bay Road, North Chicago, IL 60064 USA; 2 Robert H. Lurie Comprehensive Cancer Center, Northwestern University, Chicago, IL 60611 USA

**Keywords:** V-ATPase, Cancer, mTOR, WNT, TGF-β, Notch signaling, Autophagy, Drug resistance, Warburg effect

## Abstract

The Vacuolar ATPase (V-ATPase) is a proton pump responsible for controlling the intracellular and extracellular pH of cells. The structure of V-ATPase has been highly conserved among all eukaryotic cells and is involved in diverse functions across species. V-ATPase is best known for its acidification of endosomes and lysosomes and is also important for luminal acidification of specialized cells. Several reports have suggested the involvement of V-ATPase in maintaining an alkaline intracellular and acidic extracellular pH thereby aiding in proliferation and metastasis of cancer cells respectively. Increased expression of V-ATPase and relocation to the plasma membrane aids in cancer modulates key tumorigenic cell processes like autophagy, Warburg effect, immunomoduation, drug resistance and most importantly cancer cell signaling. In this review, we discuss the direct role of V-ATPase in acidification and indirect regulation of signaling pathways, particularly Notch Signaling.

## Background

The Vacuolar ATPase (V-ATPase) is a multi-subunit ATP driven proton pump that acidifies intracellular vesicles and extracellular milieu and thereby is involved in a large number of biological functions [1]. Previous reviews have elegantly described the structure and function of V-ATPase [2–4]. Here we review the recent literature pertaining to V-ATPase function and contribution to various cell processes in normal physiology with an emphasis on cancer. We also present in detail the regulation of Notch and other signaling pathways by V-ATPase revealing a hitherto less known function of V-ATPase in cell signaling.

### Subunit isoforms

Structurally, the V-ATPase is a rotary nanomotor made up of multiple subunits, each with multiple isoforms [5]. Subunits are arranged in two domains: a peripheral V_1_ domain, responsible for ATP hydrolysis and an integral membrane domain V_O_, which functions in proton translocation. The structure of V-ATPase has been highly conserved among all eukaryotic cells and is involved in diverse functions across species. In mammals, V_1_ domain has eight different subunits (*A, B, C, D, E, F, G and H*) whereas the V_O_ domain is composed of six different subunits (*a, c, c’, c”, d, e*) [6]. The differential requirement of acidification in intracellular vesicles and extracellular milieu drives V-ATPase function and regulation. To decrease or increase pump efficiency V-ATPase controls the coupling between ATP hydrolysis and proton pumping. This process is brought about by the *‘a’* subunit of V-ATPase [7]. Similarly, cell and compartment specific targeting of V-ATPase is also dependent on the ‘a’ subunit isoforms. V_O_*a* is a 100-kDa integral membrane protein with an N terminal cytosolic tail and 9 transmembrane domains. Four isoforms of the ‘*a*’ subunit (*a1, a2, a3 and a4*) have been identified with distinct vesicular and cell type distribution. V_O_*a*1 is expressed on the synaptic vesicles and V_O_*a2* is expressed on intracellular vesicles like golgi and early endosomes. V_O_*a*3 is expressed on plasma membrane of osteoclasts, whereas V_O_*a*4 is expressed on the plasma membrane of renal intercalated cells. Further, the N-terminus of subunit ‘a’ is an important motif that tethers V1 domain to the membrane and has also been reported to be a unique pH sensor in lysosomes [8]. The expression and isoform localization of subunit ‘a’ is critical to the functioning of V-ATPase [5].

### Physiological function of V-ATPase

The V-ATPase is ubiquitously expressed and performs diverse biological functions within cells of most tissues through vesicular, luminal and extracellular acidification [9]. To achieve numerous cellular functions, V-ATPase facilitates localized concentration of protons in acidic vesicles of the endocytic and exocytic pathways [1].

### Vesicular acidification

#### Endosomes and lysosomes

V-ATPase is best known for its role in the acidification of intracellular vesicles like endosomes and lysosomes. On the surface of endosomes, V-ATPase acidifies and thereby modulates important cellular processes like receptor endocytosis and vesicular trafficking [10]. Acidification of endosomes by V-ATPase is crucial for endocytic internalization of receptor ligand complexes. Following signaling initiation, lower pH in endosomes releases the ligand recycling it to the plasma membrane [11]. In lysosomes, V-ATPases help maintain the low pH of 4.5 and are also important for transportation of newly synthesized acid hydrolases from Golgi to lysosomes. Further, phagosomes and autophagosomes in macrophages and tumor cells respectively, also depend on the acidic pH maintained by V-ATPase for the activity of the degradative enzymes in these compartments [12].

#### Golgi

The sorting of exocytic and endocytic machinery begins at the golgi complex. Importantly, most proteins undergo glycosylation, a crucial posttranslational modification within the golgi apparatus [13]. Mutations in the a2 subunit of V-ATPase result in cutis laxa, an autosomal recessive wrinkly skin syndrome wherein impaired glycosylation of extra cellular matrix proteins is observed [14]. Although V-ATPase has been genetically correlated to glycosylation defect, the exact relationship between golgi acidification and protein maturation has not been explored.

#### Specialized vesicles

V-ATPase is a major protein expressed in specialized compartments of specific cell types. During neurotransmission, V-ATPase provides the crucial proton motive force necessary for the formation of synaptic vesicles and subsequent accumulation of neurotransmitters [15]. In pancreatic cells, V-ATPase dependent acidification is important for insulin exocytosis [16]. V-ATPase also governs the fission-fusion balance of vesicular system by interacting with Soluble NSF Attachment protein Receptor (SNARE) and GTPase [17].

### Luminal acidification

**Fig. 1:**
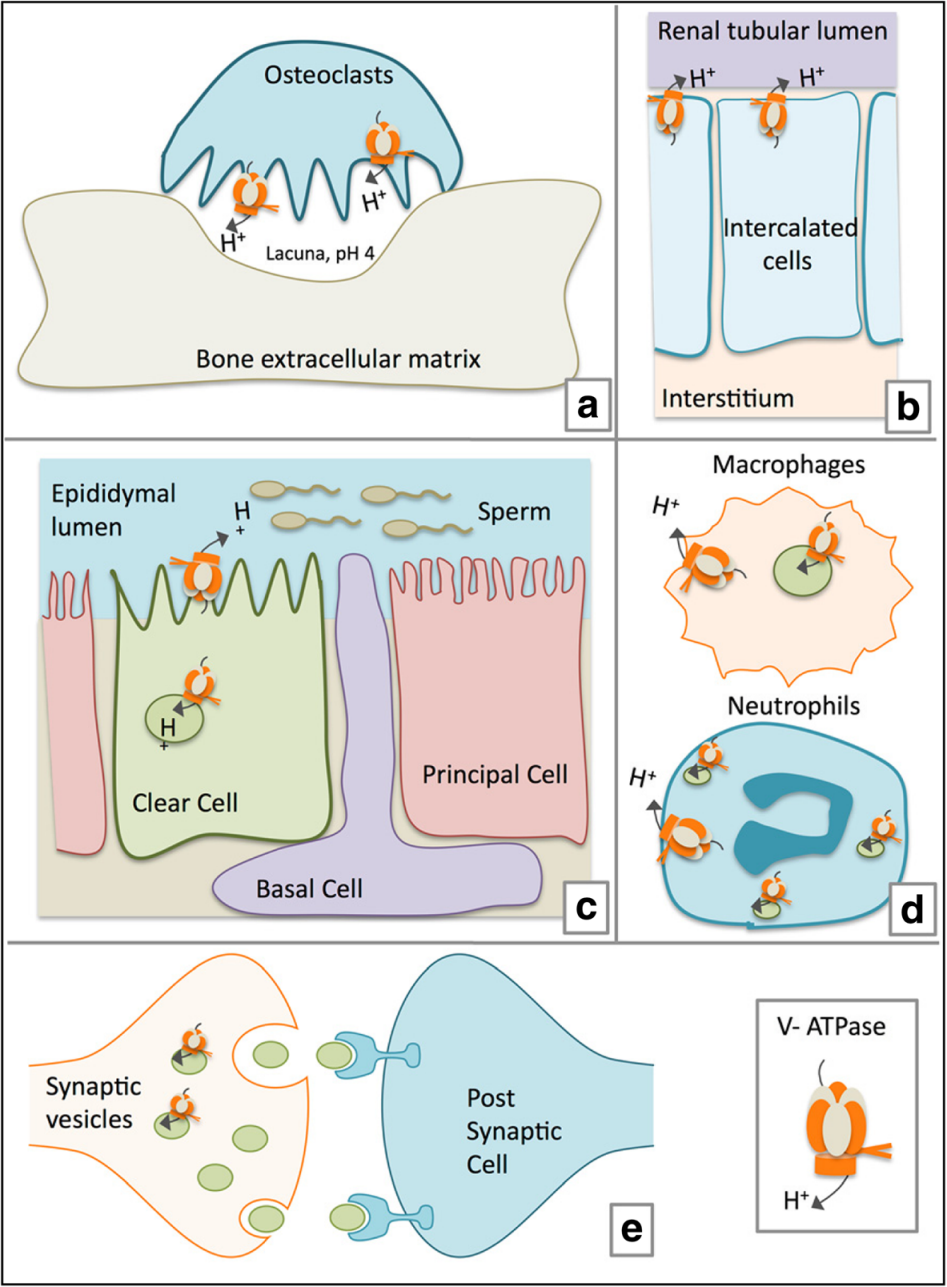
Physiological role of V-ATPase in luminal acidification. The involvement of V-ATPase is involved in numerous specialized cell processes including bone resorption, renal function, sperm maturation, innate immune responses and neurotransmission is outlined. **a** Bone resorption: V-ATPase located on the plasma membrane of osteoclasts mediates extracellular acidification for bone demineralization during bone resorption. **b** Renal function: In the kidney, intercalated cells maintain systemic acidosis and achieve urinary acidification by proton pumping activity of V-ATPases expressed on apical membrane. **c** Sperm maturation: In the epididymis, V-ATPase expressing clear cells acidify the lumen, a process that is crucial for the proper maturation and motility of spermatozoa. **d** Innate immune responses: V-ATPases mediated vesicular acidification has an important role in trafficking and exocytosis of neutrophil granules V-ATPase is constitutively expressed on the plasma membrane of monocytes and activated lymphocytes and contributes to pH related inflammatory responses. **e** Neurotransmission: V-ATPase provides the crucial proton motive force necessary for the formation of synaptic vesicles and subsequent accumulation of neurotransmitters. V-ATPase provides the crucial electrochemical potential necessary for accumulation of neurotransmitters in the secretory synaptic vesicles

V-ATPases were initially identified on intracellular vesicles, but the importance of plasma membrane V-ATPases has grown enormously [18]. In the epithelial cells of proximal tubule of kidney, the a4 isoform of V-ATPase maintains acid base balance and acidification of urine (systemic acidosis) [19, 20]. Similarly, in clear cells of the epididymis, plasma membrane V-ATPase acidifies the luminal compartment and helps in sperm maturation and storage [21, 22]. In osteoclasts of the bone, lysosomal V-ATPase translocates to the plasma membrane during bone resorption to acidify the lacunae [23]. Plasmalemmal V-ATPase is crucial to the functioning of interdental cells of the ear, epithelial cells of the nose and vision [24–26]. V-ATPase dysfunction is associated with pathological conditions like renal tubular acidosis, deafness, impairment of olfactory sense, and osteoporosis [27–29] A schematic outlining the role of V-ATPase in vesicular and luminal acidification is shown in Fig. 1.

### Role in cancers

Recently, plasma membrane V-ATPase has been extensively studied in cancer, where they help maintain an alkaline intracellular environment favorable for growth and an acidic extracellular environment favorable for invasion [30]. In tumors, V-ATPase expression has been shown to be higher towards the leading edge of proliferating cancer cells of breast, prostate, lung, ovarian, liver, pancreatic, melanoma and esophageal cancers [2]. Specifically, breast cancer cells express V-ATPase on the plasma membrane to acidify extracellular space and the quantitative expression of V-ATPase correlates with invasiveness and metastatic potential of the cell line [31]. The exact contribution of V-ATPase to the growing tumor is achieved through its influence on the molecular mechanisms/pathways discussed below.

### Immunomodulation

The *a*2 isoform of Vacuolar ATPase (V_O_*a*2 or *a*2V) has an immunomodulatory role in pregnancy and cancer. Studies involving *a*2V in reproductive biology unearthed a hitherto unknown role for this molecule in normal sperm maturation and production in addition to embryo implantation [22, 32]. In the tumor microenvironment, the N terminal domain of *a*2V polarizes macrophages to the tumor-associated macrophages (M2 type) and stimulates different monocyte subsets through endocytosis pathway [33]. Following these findings, it was further demonstrated that *a*2V deficiency in tumor cells alters the resident macrophage population in the tumor microenvironment and affects in vivo tumor growth [34]. *a*2V is expressed on the primary granules of neutrophils and helps maintain pH in exocytic pathway during neutrophil activation [35]. Treatment of human neutrophils with recombinant N terminal peptide of a2V (a2NTD) promoted neutrophil migration and polarization [36]. Together, these studies highlight the immunomodulatory role of V-ATPase in eliciting potent immune responses.

### Warburg effect

A hallmark of cancer is the Warburg effect where cells shift from oxidative phosphorylation to aerobic glycolysis [37]. Several studies point to the hypothesis that cancer cells depend on V-ATPase more than any other pH regulators like Na^+^H^+^ exchangers, bicarbonate transporters and proton-lactate symporters to achieve the favorable alkaline intracellular pH and acidic extracellular pH [38]. Alkalization of cytosol activates glycolysis while suppressing oxidative phosphorylation [39]. Further, some glycolysis related oncogenes like Hypoxia Induced Factor (HIF-1) are regulated by pH alteration induced by V-ATPase [40].

### Acid proteases

Consequent to extracellular acidification in tumors is the activation of acid proteinases, which are enzymes that cleave the extracellular matrix during tumor invasion. These enzymes belong to the class of acid proteinases like cathepsins [41], Matrix Metallo Proteinases (MMP) and gelatinases and are active at acidic pH [42, 43]. Furthermore, the activity of intracellular enzymes like γ-secretase, which are active at acidic pH, is also enhanced with increased activity of V-ATPase in vesicles [44]. Subsequently, this leads to dysregulation of oncogenic pathways like Notch.

### Drug resistance and V-ATPase inhibitors

Altered pH of tumor microenvironment may influence sensitivity to chemotherapeuticdrugs [45]. Anthracyclines and alkaloids have a pKa of 7 to 8 and are internalized to the endosomal compartment [46]. Recent data suggests that use of V-ATPase inhibitors not only causes cytosolic pH alterations leading to cell death but also enhances drug uptake, thereby making an effective component of combinatorial treatment to cancer [47]. In ovarian cancer, *a*2V is expressed on the leading edge of cancer cells and modulates the activity of MMP9. Further, *a*2V contributes in cisplatin mediated drug resistance in ovarian cancer and selective inhibition of *a*2V could serve as an efficient strategy to treat chemo-resistant ovarian cancer [48]. The V-ATPase inhibitors bafilomycin and concanamycin belong to a class of pleomacrolides that target the V_O_ sector and efficiently inhibit V-ATPase activity. Recently, Apicularen and archazolids have been reported to be potent and specific inhibitors of V-ATPase [49]. However, all available small molecule inhibitors have significant toxicity considering the involvement of V-ATPase in normal cell physiology [50]. Therefore development of specific neutralizing antibodies against the ‘*a*’ subunit isoform that has cell specific expression could be an efficient alternative to cause direct V-ATPase inhibition while also tackling multi drug resistance indirectly with combinatorial use [51].

### Autophagy

Autophagy is the process of selective degradation or recycling of cargos delivered by autophagosomes to lysosomes [52]. Tumor cells show varied dependence on autophagy as they progress from primary tumor to the highly metastatic solid tumor [53]. Cellular cargo marked for degradation are delivered to the lysosomes by autophagic processes. The proton pumping activity of V-ATPase is responsible for activation of lysosomal acid hydrolases which degrade cargo uptake from autophagosomes [54]. Although studies point to the requirement of functional V-ATPase for autophagy [55] and V-ATPase inhibitor Bafilomycin is used as classic inhibitor of autophagy [56], the exact role of V-ATPase in membrane dynamics of autophagic flux is not understood. A recent study reported that treatment with Bafilomycin, which inhibits the activity of both V-ATPase and Ca^2+^ pump SERCA pump led to blockade in autophagic flux whereas V-ATPase deficient lysosomes were still capable of fusing with autophagosomes [57]. These results suggest the involvement of V_ATPase in degrading autophagic cargo in lysososomes than in autophagic flux and highlight the need for developing specific inhibitors and gene manipulation techniques to study the exact role of V-ATPase in various important cell processes.

### Signaling

The endolysosomal pathway is important for both positive and negative regulation of signaling pathways [8, 58]. The first known report of involvement of V-ATPase in signaling came from a study showing that inhibition of V-ATPase by Bafilomycin affected internalization of EGFR [59]. Since then, V-ATPase has been associated with signal transduction [60] associated with m-TOR (mammalian Target Of Rapamycin), Wnt, TGF-β and Notch Signaling regulation.

### Notch signaling

Perhaps the most well studied signaling pathways regulated by V-ATPase is Notch. This can be attributed to the fact that Notch signaling depends on the endolysosomal pathway for its activation, maintenance and degradation of key pathway mediators [61–63]. V-ATPase maintains cellular pH balance and plays an important role in endocytosis, protease activation and protein degradation. Specifically, *a*2V (V-ATPase subunit- V_O_*a*2) was previously localized to early endosomes - the site for receptor endocytosis [8]. Following ligand binding, Notch receptor takes the endocytic route and is cleaved by proteases for activation. Later, the receptors are degraded in the lysosome [63]. In Drososphila, mutations in Vps25, a component of ESCRT machinery that regulates endosomal sorting of signaling receptors, causes accumulation of the Notch receptor in endosomes and enhances Notch signaling [64]. In a study analyzing drosophila mutations of Hrs, another component of ESCRT, Notch accumulates in endosomes but does not cause ectopic activation of Notch signaling [65]. The loss of autophagy leads to activation of the Notch signaling in the Drosophila ovarian follicle cells due to disruption of Notch degradation [66]. Contrary to these reports, an independent study found that mutations in Rabconnection-3 disrupt the proton-pumping activity of V-ATPase and accumulate Notch in late endosomes after S2 cleavage, thereby reducing Notch Signaling in Drosophila and mammalian cells [67]. These findings were followed by reports in Drosophila further indicating that through the acidification of endolysosomal pathway, V-ATPase is required for the activation of Notch in endosomes as well as for the degradation of Notch in lysosomes [68]. During mammalian development, expression of a dominant negative subunit of V-ATPase in neural precursors reduced Notch signaling and depleted neural stem cells leading to neuronal differentiation [69]. Recently, studies in astrocytes in the retina of Nuc1 mutated rats were shown to dysregulate Notch signaling. The reduction in Notch signaling was due to mutated βA3/A1-crystallin, which regulates V-ATPase activity resulting in impaired endosomal acidification and γ-secretase activity thereby affecting the rate of Notch receptor processing [70]. This is an interesting finding considering that the role of V-ATPase in vision in now emerging [26]. Together these findings indicate that the regulation of Notch signaling by V-ATPase can have both positive and negative outcomes depending on the cellular localization of V-ATPase activity affected (endosomes vs lysosomes) and the dependence of Notch receptor processing on the endosomal pathway [71, 72]. Although the V-ATPase and Notch crosstalk has been investigated in the context of V-ATPase dependent endolysosomal acidification affecting Notch signaling, a recent report suggests that regulation could also be vice-versa. Specifically, the authors suggest that Presinilin1 (PS1), a component of the γ-secretase enzyme complex responsible for cleavage of Notch receptor and β-amyloid peptide physically interacts with the V_O_*a*1 isoform of V-ATPase and targets it from the endoplasmic reticulum to the lysosomes [73]. Our studies have identified that V-ATPase regulates Notch Signaling in breast cancer [74] and mammary gland development [75]. *a*2V is expressed on the surface of proliferating mammary epithelial cells and Triple Negative Breast Cancer (TNBC) cells, indicating its role in cell proliferation during normal development and disease. In TNBC, *a*2V inhibition enhances Notch Signaling by blocking lysosomal and autophagic degradation of Notch receptor [74]. Loss of *a*2V in mouse mammary gland leads to abnormal Notch activation and impairs ductal morphogenesis, causing lactation defects [75]. Notch signaling is activated during preterm labor induced by infection with PGN + poly (I:C), resulting in upregulation of pro-inflammatory responses, and its inhibition improves in-utero survival of live fetuses. Further in preterm labor induced by inflammatory response to LPS injection, up-regulation of Notch-related inflammation and down-regulation of angiogenesis factors was observed [76]. In both infection and inflammatory preterm labor models, we were able to rescue the phenotype by treating with γ-secretase inhibitors (GSI) [77]. This paves a way for important future direction especially since GSI is an efficient inhibitor of Notch Signaling and is currently in clinical trials for several cancers. With this, the V-ATPase and Notch crosstalk emerges to be important during normal development and indiseases like Alzheimers and various cancers [78].

### Wnt signaling

The Wnt signaling pathway plays a major role in cell and tissue maintenance, polarity and differentiation. In humans, dysregulation of Wnt signaling has been implicated in cancer [79]. A classic example of dysregulated Wnt signaling is colorectal cancer wherein the loss of Adenomatous Polyposis Coli (APC), a negative regulator of Wnt signaling triggers tumorigenesis [80]. During signaling, Wnt ligands act on target cells by binding to Frizzed, Fz and LRP (low density-lipoprotein,) a cell surface receptor complex leading to disassembly of Glycogen Synthase Kinas (GSK-3) and subsequent release β-catenin. β-catenin is the main downstream mediator of Wnt pathway, which activated Wnt target oncogenes genes like c myc and cyclinD1 [81]. The (P) RR, Pro Renin Receptor also called ATP6ap2 acts as an adaptor molecule between V-ATPase and Wnt receptor complex LRP 5/6 [82]. In Xenopus and Drosophila, it has been shown that V-ATPase interacts with LRP 5/6 receptor complex and both genetic knockdown and pharmacological inhibition of V-ATPase interfere with signal transduction and significantly reduce cellular response to Wnt signaling [83, 84]. Furthermore, V-ATPase indirectly regulates Wnt signaling mediator β-catenin and Notch mediator NICD has been demonstrated through autophagy [85].

### TGF-β signaling

Mutations in the a2V gene cause Autosomal recessive Cutis Laxa (ACL) syndrome where patients present with decreased amount of extra cellular matrix proteins like Collagen resulting in wrinkly skin phenotype [86]. Supporting theses findings, a mechanistic investigation of the mutations responsible for cutis laxa in humans identified *a2*^P405L^ mutation to be unstable and defective in golgi trafficking compared to wild type [87]. Further, reports point to a glycosylation defect in ACL resulting in elevated promotes transforming growth factor-beta (TGF-β) signaling in these patients with *a*2V mutations [88]. V-ATPase promotes TGF-β induced epithelial-mesenchymal transition of rat proximal tubular epithelial cells [89]. In addition to its effect on Notch Signaling, *a*2V inhibition activated Wnt pathway in TNBC and TGF-β pathway in mammary epithelial cells [75]. This suggests that the role of *a*2V in modulating signaling mediators is not exclusive to Notch. Further, these mice also displayed a reduction of total collagen due to impaired glycosylation [90].

### mTOR signaling

In mTOR signaling, the Serine threonine kinase mTOR and other components of the mTOR complex 1 (mTORC1) sense amino acid availability cellular stress, and modulate growth [91] .Upon amino acid stimulation, V-ATPase activates Guanine Exchange Factor (GEF) activity of Ragulator towards RagA which in turn promotes RagC GTP hydrolysis [92]. The GTP-bound RagA and GDP-loaded RagC together recruit mTORC1 to the lysosomal surface [93]. Activated mTORC1 responds to growth factor signaling controls the regulatory switch from cell death to proliferation [94]. A recent report suggested the involvement of osteoclast proton pump regulator Atp6v1c1 in enhancing breast cancer growth by activating the mTORC1 pathway and bone metastasis by increasing V-ATPase activity [95].

## Conclusions

**Fig. 2:**
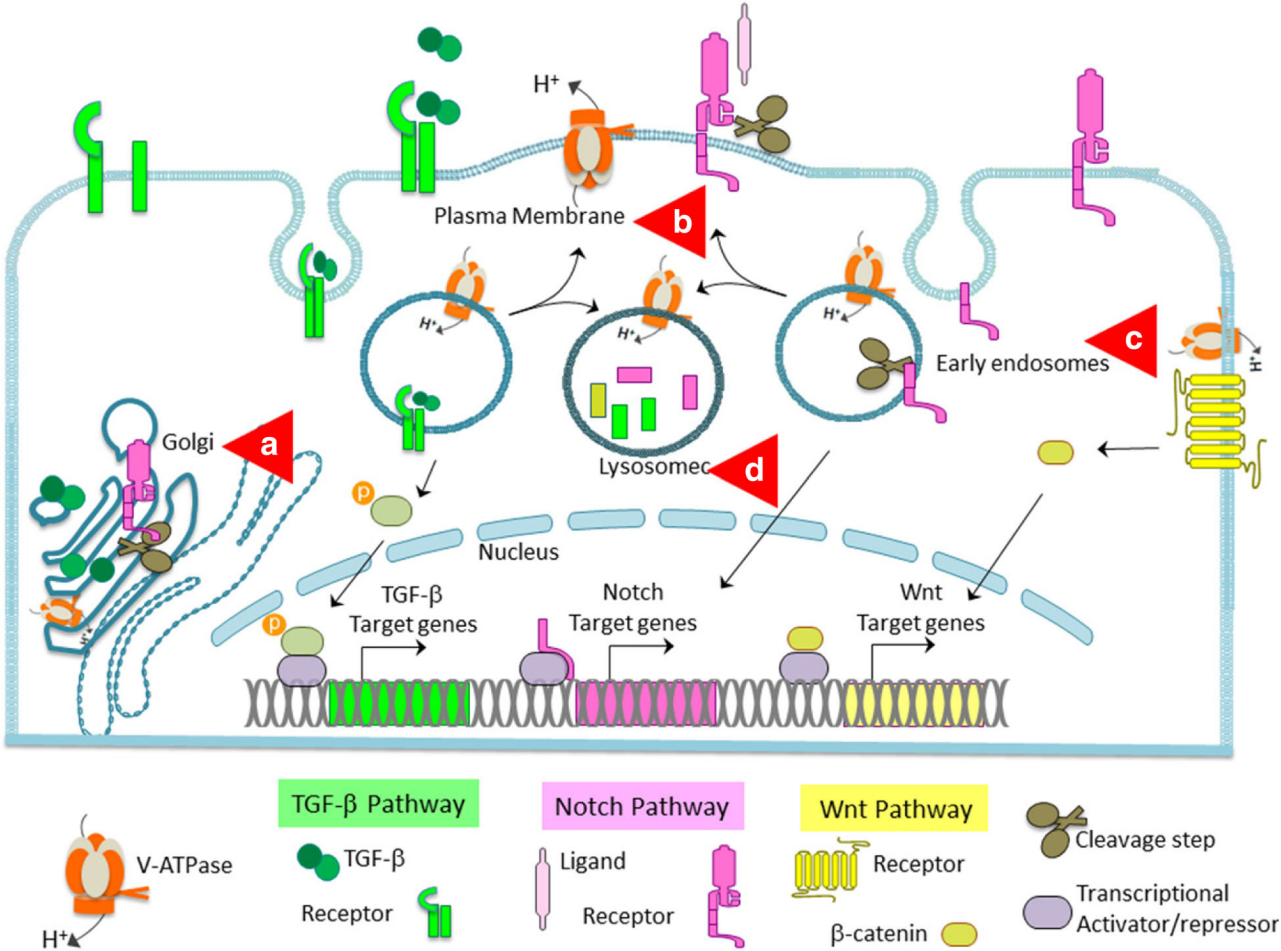
The mechanistic basis of V-ATPase dependent signaling. V-ATPase (orange) acidifies intracellular vesicles thereby regulating Notch signaling and other pathways like Wnt and TGF-β, which depend on endolysosomal system for sustenance. In Notch Signaling, the Notch receptor (dark pink) is cleaved in Golgi and translocated to the plasma membrane where further cleavage of the receptor occurs in response to Notch ligand (light pink) binding. Cleaved Notch intracellular domain is translocated to nucleus activating Notch target genes. TGF-β (dark green) protein is glycosylated in the Golgi to form mature TGF-β and secreted into the extracellular space. TGF-β bound to its receptor (TGF-βR) (bright green) results in endocytosis and phosphorylation of Smad2 (olive green), which in turn activates TGF-β target genes. During canonical Wnt signaling, the binding of ligands to the Wnt receptor complex (bright yellow) inhibits the phosphorylation of β-catenin (dark yellow) by GSK-3β and directs the translocation of β-catenin into the nucleus where it activates the transcription of target genes Cyclin D1 and oncogene c-Myc. V-ATPase-mediated acidification can affect signaling in the following ways: **a** Maturation of signaling molecules Notch receptor and TGF-β by furin glycosylation in the golgi vesicles. **b** Cleavage and activation of pathway mediators by acid-dependent enzyme like matrix metallo proteinases (MMPs) and γ-secretase. **c** Maintenance of basal signaling by recycling endocytosis of both ligand and receptor. **d** Degradation of signaling molecules in lysosomes

Most studies until now have focused on the endolysosomal component of V-ATPase acidification and associated activation/degradation of signaling mediators. The signaling pathways identified to be associated with V-ATPase namely Notch, Wnt and TGF-β surprisingly share similar expression patterns and cellular functions during both development and disease. However, there are other steps of the signaling pathways known to be pH dependent, which warrant future investigation. Maturation of Notch and TGF-β by glycosylation in Trans Golgi Network (TGN) activates their signaling pathways. Further *a*1 and *a*2 subunits of V-ATPase are important for the protein glycosylation that is a key role of the TGM. [8]. We and others have shown that surface expression of V-ATPase modulates MMPs thereby leading the proliferation ofcancer cells [43, 48]. However, V-ATPase dependent activity of ADAM/TACE has not been explored and might hold important clues for V-ATPase and signaling crosstalk mechanism. Furthermore, enzymes like γ-secretase that activate signaling pathway mediators are efficient at acidic pH [44]. Similarly, the involvement of V-ATPase in activation of acid proteases during lysosomal degradation to regulate signal turnover cannot be ignored [96] (See summary Fig. 2). V-ATPase could have profound effects on cell fate by influencing signaling molecules that depend on pH. The research on V-ATPase regulation of signaling pathways is a field waiting to be explored that will have a tremendous impact in physiology and pathology.
